# Association of CpG-SNP and 3'UTR-SNP of WFS1 with the Risk of Type 2 Diabetes Mellitus in an Iranian Population

**DOI:** 10.22088/BUMS.6.4.197

**Published:** 2017-11-21

**Authors:** Shahram Torkamandi, Milad Bastami, Hamid Ghaedi, Shahriar Tarighi, Fazlollah Shokri, Abdolreza Javadi, Reza Mirfakhraie, Mir Davood Omrani

**Affiliations:** 1 *Department of Medical Genetics, Faculty of Medicine, Shahid Beheshti University of Medical Sciences, Tehran, Iran.*; 2 *Department of Medical Genetics and Immunology, Faculty of Medicine, Urmia University of Medical Sciences, Urmia, Iran.*; 3 *Department of Medical Genetics, Faculty of Medicine, Tabriz University of Medical Sciences, Tabriz, Iran.*; 4 *Department of Medical Laboratory Sciences, School of Allied Medical Sciences, Shahid Beheshti University of Medical Sciences, Tehran, Iran.*; 5 *Department of Pathology, * *Imam Hossein Hospital, Shahid Beheshti University of Medical Sciences, Tehran, Iran.*

**Keywords:** WFS-1, type 2 diabetes, genome wide association study, single nucleotide polymorphisms

## Abstract

Type 2 diabetes mellitus (T2DM) is one of the most common multifactorial disorders in Iran. Recent genome wide association studies (GWASs) and functional studies have suggested that *WFS1* may predispose individuals to T2DM. However, to date, the possible association of such variants with T2DM in Iranians remained unknown. Here, we investigated the association of the two polymorphisms of *WFS1* (rs1801214 a CpG-SNP, and rs1046320 a 3’UTR-SNP) with T2DM in an Iranian population. The study population comprised 432 unrelated Iranian individuals including 220 patients with T2DM, and 211 unrelated healthy control subjects. Genotyping was performed using PCR-RFLP, and confirmed with sequencing. In a logistic regression analysis, the rs1801214-T allele was associated with a significantly lower risk of T2DM assuming the log-additive model (OR: 0.68, 95% CI: 0.52-0.91, P= 0.007539). Moreover, the G allele of rs1046320 was associated with a lower risk of T2DM assuming the log-additive model (OR: 0.68, 95% CI: 0.50- 0.91, P= 0.008313). Haplotype analysis revealed that haplotypes that carry at least one protective allele are associated with a lower risk of T2DM. This is a first evidence for the association of* WFS1* rs1801214, and rs1046320 with T2DM in an Iranian population.

type 2 diabetes mellitus (T2DM), the seventh cause of worldwide mortality is a complex disorder resulting from the absolute or relative lack of insulin (1). Prevalence of diabetes is greater than 14% in Iranians aged above 30 years, and according to the International Diabetes Federation (IDF) website, more than 4.5 million Iranians were diagnosed as diabetic cases in 2014 (1,2). Sufficient insulin secretion from pancreas beta cells is essential to maintain blood glucose within normal range, and destroy beta cells during T2D development upon insufficient insulin secretion and diabetes progression (3,4).

A variety of environmental, epigenetic, genetic factors. and also interplay between these factors contribute to the development of T2DM. Genetic susceptibility to T2DM has been extensively explored by genome- wide association studies (GWASs), and accordingly a number of loci have been identified to be associated with T2DM (5-7). Among these, variants in the Wolferamin (*WSF1*) have been shown to modify the susceptibility to T2DM in different studies (8, 9).

Wolferamin is a 890 amino-acid transme-mbrane polypeptide located on chromosome 4p16 that is ubiquitously expressed, but has high expression rate in specific neurons and pancreatic beta cells (10). This protein is a novel endoplasmic reticulum (ER) transmembrane glycoprotein that plays a role in regulating calcium fluxes, and hemostasis in the ER (11,12). Mutations in *WFS1* are known to cause the Wolfram syndrome (WFS; OMIM 222300), an autosomal recessive neurod-egenerative disorder that is clinically defined by diabetes insipidus, young-onset non-immune insulin-dependent diabetes mellitus, optic atrophy, and deafness. Therefore, WFS is also known as DIDMOAD syndrome (13). A growing body of evidence suggested that highly developed ER structure in beta cells had important roles in insulin production, and secretion in response to blood glucose levels (14-17). Impaired insulin secretion due to epigenetic modifications, and pathogenic variants in the *WSF1* results in progressive rodent and human glucose intolerance, and insulin deficiency due to ER stress and apoptosis of beta cells (11,18,19). Polymorphisms of the *WFS1* was suggested as a minor modulator of gene function, and susceptibility to polygenic forms of diabetes. To the best of our knowledge, there are no data regarding the possible contribution of the GWAS-identified locus at this gene to T2DM in Iranian population.

In this study, we evaluated the association of two *WFS1* variants with T2DM in an Iranian population. These variants include *WFS1* rs1801214, an index variant of the GWAS identified locus that removes a cytosine-phosphate-guanine (CpG) dinucleotide, and rs1046320 at 3’UTR of *WFS1* that is a candidate functional variant in high linkage disequilibrium (*r*^2^= 0.97) with the index variant.

## Materials and methods

**Subjects**

The study population comprised 432 unrelated Iranian individuals, including 220 patients with T2DM and 211 unrelated healthy control subjects, matched for age and gender. The diagnosis of T2DM was based on the WHO criteria (20, 21). T2DM was diagnosed as fasting plasma glucose (FPG) levels of ≥ 126 mg/dl, and 2-hour glucose concentrations of ≥ 200 mg/dl after a 75 g oral glucose tolerance test or HbA_1c_ > 6.5%. Written informed consent was taken from all participants. This study was approved by the Ethics Committee of Shahid Beheshti University of Medical Sciences, Control subjects were those without any past documented history of glucose intolerance or family history of diabetes that have glucose concentrations below the thresholds for T2DM.

**DNA extraction**

Peripheral blood samples were collected in EDTA tubes, and transferred to Medical Genetics department laboratory for DNA extraction. The DNA was extracted by use standard salting out method. The quality and quantity of extracted DNA was measured spectrophotometri cally at 260nm/

280 nm wave length.

**Genotyping**

Genotyping was performed using polymerase chain reaction followed by restriction fragment length polymorphism (PCR-RFLP) analysis (22). The PCR reaction consisted of 30 cycles at annealing 64 C for rs1801214 and 60 C for rs1046320. Restriction enzymes and primer sequences are presented in table 2. To confirm the accuracy of the genotyping method, twenty samples were randomly selected and the assigned genotypes were confirmed by Sanger sequencing of the genomic regions encompassing the polymorphisms (Table 2).

**Statistical analysis**

Genotyping results were tested for significant departure from Hardy-Weinberg equilibrium among patients and controls using χ^2 ^and all statistical analyses were performed by R programming language (version 3.1.0) (23). Differences in clinical variables and demographic characteristics between patients and controls were evaluated using *t*-test for continuous variables and χ^2^ for categorical variables. Multivariate logistic regression analysis was undertaken in order to control age, sex and BMI category. The association of rs1801214, and rs1046320 with T2DM was implemented in the SNPassoc package (version 1.9-2). Odds ratios (OR), and respective 95% confidence intervals (95% CI) were calculated considering codominant, dominant, recessive, overdominant, and log-additive models. A p value <0.05 was considered to be statistically significant in this study .

**Table 1:** Characteristics of the study population

	**Patients (N=220)**	**Controls (N=211)**	**P value**
**Age (years)/Median**	61.78 ± 9.71	60.71 ± 8.50	0.2396
**Sex, Male (%)**	43.69	46.44	-
**BMI (Kg/m** ^2^ **)**	31.01 ± 4.79	± 1.68	**> 0.001**
**FPG (mg/dl)**	148.36 ± 27.50	88.47 ± 9.69	**> 0.001**

BMI: body–mass index; FPG: fasting plasma glucose. Quantitative variables are shown as mean ± standard deviation, and qualitative variable is shown as percent. P values less than 0.05 are shown in bold face, and are statistically significant

**Table 2.** The primer sequences of RFLP, sequencing, and PCR conditions for the studied SNPs

**SNP**	**Type of primers**	**Primer sequences** **5’ 3’**	**Enzyme**	**PCR condition (** ^o^ **C/s )**			**DNA/ fragment size (bp)**
				**Den.**	**Ann.**	**Ext.**	
rs1801214	PCR-RFLP	F:TTAGCCACCTGGTCGTCGTCAA	Hind II	95/30	64/30	72/30	123/106+17
		R:AGGGCACAAGGTAGCAGTAGGTGC					
	Sequencing	F:GCAACCTCACCATCGACTTC		95/30	59/30	72/30	600
		R:AGGGCACAAGGTAGCAGTAG					
rs1046320	PCR-RFLP	F:CTTTCACCAGTGCCGCCTGTG	*Sph I*	95/30	60/30	72/30	96/77+19
		R:GTATTCCCTTTGTCGGGGTGCA					
	Sequencing	F:GATCGAGTTCAGCACCATCCT		95/30	60/30	72/30	679
		R:AAAGGGGAAGAGCTGCTAAGG					

Den: denaturation; Ann: annealing; Ext: extension.

## Results

**Population characteristics**

Clinical characteristics and demographic data are shown in table 1. The patients and controls were matched for age, and sex in this study. The patients had higher levels of BMI and FPG than those of controls (Table 1).

**Association of** ***WFS1*** **polymorphisms with T2DM**

The genotype frequencies of rs1801214, and rs1046320 were not significantly deviated from Hardy–Weinberg equilibrium among controls (P= 0.42 and 0.24, respectively). The genotype frequencies of the studied SNPs in patients and controls are presented in table 3. *WFS1* rs1801214 was associated with T2DM assuming codominant, recessive and log-additive modes of inheritance (Table 3). In the codominant model, individuals carrying the TT genotype had a significantly lower risk of T2DM in comparison with those who carry the CC genotype (TT vs. CC, OR: 0.41, CI: 0.22-0.77, P*=*0.016909). In the recessive model, individuals carrying the TT genotype had a lower risk of T2DM in comparison with those with CC+CT genotypes (TT vs. CC+CT, OR: 0.47, CI: 0.26 – 0.83, P= 0.008355). However, the model with the lowest P value was log-additive (OR: 0.68, CI: 0.52-0.91, P= 0.007539).

Logistic regression analysis revealed that rs1046320 was associated with T2DM risk in different modes of inheritance. In codominant model, individuals carrying the GG genotype had a lower risk of T2DM in comparison to those with AA genotype (OR: 0.47, CI: 0.23-0.93*, *P= 0.030530). The association was also statistically significant assuming a dominant model (AG+GG vs AA, OR: 62, CI: 0.42- 0.91*, *P=0.014049), recessive model (GG vs AA+AG, OR: 0.55, CI: 0.28-1.08, P= 0.078039), over dominant model (AG vs AA+GG, OR: 0.74, CI: 0.50- 1.10, P= 0.136481) or a log additive model (OR: 0.68, CI: 0.50- 0.91, P= 0.008313). The log-additive model had the lowest P value (Table 3).

**Table 3:** The distribution of genotypes in T2DM cases and controls

**Model**	**Genotypes**	**Patients** **N(%)**	**Controls** **N (%)**	**OR** **(95% CI)**	**P **
**rs1801214**					
Codominant	CC CT TT	102 (45.9) 100 (45.0) 20 (9.0)	78 (37) 96 (45.5) 37 (17.5)	1.00 0.80 (0.53-1.20) 0.41 (0.22-0.77)	0.016909
Dominant	CC	102 ( 45.9)	150 (50.0)	1.00	0.057837
	CT+TT	120 (54.1)	78 (37)	0.69 (0.47-1.01)	
Recessive	CC+CT	202 ( 91.0)	174 (82.5)	1.00	0.008355
	TT	20 (9.0)	37 (17.5)	0.47 (0.26– 0.83)	
Overdominant	CC+TT	122 ( 55.0)	115 (54.5)	1.00	0.924656
	CT	100 (45.0)	96 (45.5)	0.98 (0.67–1.43)	
log-Additive	-	-	-	0.68 (0.52-0.91)	**0.007539**
**rs1046320**					
Codominant	AA	134 (60. 4)	100 (48.5)	1.00	0.030530
	AG	73 (32.9)	82 (39.8)	0.66 (0.44- 1.00)	
	GG	15 (6.8)	24 (11.7)	0.47 (0.23-0.93)	
Dominant	AA	134 (60.4)	100 (48.5)	1.00	0.014049
	AG+GG	88 (39.6)	106 (51.5)	0.62 (0.42-0.91)	
Recessive	AA+AG	207(93.2)	182 (88.3)	1.00	0.078039
	GG	15 (6.8)	24(11.7)	0.55 (0.28-1.08)	
Overdominant	AA+GG	149 (67.1)	124 (60.2)	1.00	0.136481
	AG	73 (32.9)	82 (39.8)	0.74 (0.50-1.10)	
log-Additive	-	-	-	0.68 (0.50-0.91)	**0.008313**

CI: confidence interval; N/A: not applicable; OR: odds ratio. P values for the most probable genetic models are indicated with bold face, and are statistically significant.

**Table 4:** Haplotype analysis of rs1801214, and rs1046320 in Iranian population

**Haplotype **	**rs1801214-rs1046320**	**Haplotype Frequency**	**OR (95% CI)**	**P value**
1	[C;A]	0.4028	1.00	
2	[C;G]	0.2374	0.48 (0.32-0.70)	**0.0002**
3	[T;A]	0.3250	0.48 (0.33- 0.70)	**0.0002**
4	[T;G]	0.0348	**0.35 (0.14- 0.88)**	**0.0252**

OR: odds ratio. P values less than 0.05 are shown in bold face. Bold values are statistically significant

**Haplotype analysis**

We further estimated the frequencies of haplotypes for these SNPs, and evaluated the association of haplotypes with T2DM. Table 4 represents the frequency of haplotypes in the studied population along with the results of association analysis. The most frequent haplotype (haplotype 1 in the table 4, consisting of rs1801214-C, and rs1046320-A) was used as a reference haplotype. The results showed that haplotypes that contain at least one protective allele (either rs1801214-T or rs1046320-G) had a lower risk of T2DM in comparison to the reference haplotype (Table 4).

## Discussion

T2DM is one of the most worldwide health problems of the 21^th^ century, and many genetic variants were shown to be associated with disease susceptibility. *WFS1* is a new susceptible diabetes gene, which has been confirmed recently with genome wide association, and replication studies (8, 9, 24-26). We showed that rs1801214 was significantly associated with T2DM in the studied Iranian population. Moreover, this is the first replication study for this SNP and the results were in line with the original GWAS with the T allele being associated with a lower risk of T2DM (8). Subjects with at least one T allele had a lower risk of T2DM. *WFS1* rs1801214 is located in the coding sequence of *WFS1*, removing a CpG dinucleotide site that has the potential for DNA methylation. It has been shown that different methylation pattern of CpGs overlapping with SNP may affect the expression of target genes (27). Analyzing 19 CpG overlapping SNPs that were identified by T2DM GWASs, Dayeh et al. showed that all CpG-SNPs were associated with different DNA methylation pattern of CpG sites at islet tissue of diabetic donors. Furthermore, they demonstrated that rs1801214 was associated not only with differential DNA methylation of surrounding CpG sites, but also with differential exon expression, and alternative splicing (19). Therefore, this genetics-epigenetics interaction may interfere with DNA binding of regulatory proteins that, in turn, may influence local DNA methylation or alternative splicing, and expression of target genes (27).

Rs1046320 is located at the 3’UTR of *WFS1* that may affect miRNA binding site, and has been another investigated variant in this study that was found to be significantly associated with T2DM risk. Previous studies demonstrated that genetic variations within or proximal to miRNA binding sites in target genes have the potential to either destroy or create miRNA binding sites which affect on target genes expression, phenotypes or causes disease (28). We identified that GG genotype or the G allele of rs1046320 had protective effect against T2DM. Several studies have been performed to elucidate the role of rs1046320 SNP in T2DM pathogenesis. Katherine et al. has performed a fine mapping experiment in UK and showed that rs1046320 has a protective effects against T2DM. This protective effect was also confirmed in a meta-analysis performed by the same authors (24). Another study on Hungarian population has evaluated the association of *WFS1* 3’UTR variants potentially affecting miRNA binding including rs1046320 with T2DM, and confirmed the protective role of the G allele (25). Besides, rs1046320 may potentially alter miR-204-5p and miR-211-5p binding to *WFS1* transcript that may imply a mechanism by which this SNP contribute to T2DM risk.

Furthermore, we estimated the frequencies of haplotypes for these SNPs and evaluated the association of haplotypes with T2DM, and identified that haplotypes carrying at least one protective allele of either SNPs were associated with a lower risk of T2DM. Different experimental and meta-analysis studies have investigated the association of* WFS1* variants located at this haplotype (rs10010131, and rs6446482) with the risk of T2DM and many lines of evidence indicate that polymorphisms of *WFS1* had important effects on insulin secretion, insulin sensitivity and the risk of hyperglycemia in T2DM which need to be confirmed in different functional analysis studies (29-31).

In conclusion, we have replicated the association between two SNPs of *WFS1*, and risk of T2M in an Iranian case-control study. Our results are a first report of significant association of the T allele of rs1801214 polymorphism and G allele of rs1046320 polymorphism with reduced risk of T2DM in Iranian population, and also a first replication study of rs1801214. Further studies are needed to fully elucidate the role of *WFS1* SNPs in T2DM susceptibility. 
